# Rationale and Safety Assessment of a Novel Intravaginal Drug-Delivery System with Sustained DL-Lactic Acid Release, Intended for Long-Term Protection of the Vaginal Microbiome

**DOI:** 10.1371/journal.pone.0153441

**Published:** 2016-04-19

**Authors:** Hans Verstraelen, Chris Vervaet, Jean-Paul Remon

**Affiliations:** 1 Department of Obstetrics & Gynaecology, Faculty of Medicine and Health Sciences, Ghent University, Ghent, Belgium; 2 Department of Pharmaceutics, Laboratory of Pharmaceutical Technology, Faculty of Pharmaceutical Sciences, Ghent University, Ghent, Belgium; University of Pittsburgh, UNITED STATES

## Abstract

Bacterial vaginosis is a prevalent state of dysbiosis of the vaginal microbiota with wide-ranging impact on human reproductive health. Based on recent insights in community ecology of the vaginal microbiome, we hypothesize that sustained vaginal DL-lactic acid enrichment will enhance the recruitment of lactobacilli, while counteracting bacterial vaginosis-associated bacteria. We therefore aimed to develop an intravaginal device that would be easy to insert and remove, while providing sustained DL-lactic acid release into the vaginal lumen. The final prototype selected is a vaginal ring matrix system consisting of a mixture of ethylene vinyl acetate and methacrylic acid-methyl methacrylate copolymer loaded with 150 mg DL-lactic acid with an L/D-lactic acid ratio of 1:1. Preclinical safety assessment was performed by use of the Slug Mucosal Irritation test, a non-vertebrate assay to evaluate vaginal mucosal irritation, which revealed no irritation. Clinical safety was evaluated in a phase I trial with six healthy nulliparous premenopausal volunteering women, with the investigational drug left in place for 7 days. Colposcopic monitoring according to the WHO/CONRAD guidelines for the evaluation of vaginal products, revealed no visible cervicovaginal mucosal changes. No adverse events related to the investigational product occurred. Total release from the intravaginal ring over 7 days was estimated through high performance liquid chromatography at 37.1 (standard deviation 0.9) mg DL-lactic acid. Semisolid lactic acid formulations have been studied to a limited extent in the past and typically consist of a large volume of excipients and very high doses of lactic acid, which is of major concern to mucosal safety. We have documented the feasability of enriching the vaginal environment with pure DL-lactic acid with a prototype intravaginal ring. Though the efficacy of this platform remains to be established possibly requiring further development, this approach may offer a novel avenue to modulate and protect the vaginal microbiota.

**Trial Registration:**

U.S. National Institutes of Health ClinicalTrials.gov NCT02314429

## Introduction

Dysbiosis of the human vaginal microbiome has long been recognized and is basically understood as a prolonged shift away from a *Lactobacillus*-dominated microbiota [1]. While encompassing different community states deplete of lactobacilli [1], our understanding of the pivotal role of the vaginal microbiome in health and disease primarily derives from a vast body of evidence on adverse health outcomes associated with bacterial vaginosis (BV), a community state characterized by a highly diverse, anaerobic microbiota thriving in a drastically different vaginal environment [2,3].

Historically, BV has received ample attention as a clinical entity. Though most often asymptomatic [4], symptomatic BV still is a leading cause of vulvovaginal complaints, typically involving profuse, foul-smelling vaginal discharge. BV symptoms often resolve spontaneously, however also frequently tend to recur over an extended period of time [5,6], negatively impacting on women’s intimate, social, and professional relationships [7,8]. Compelling epidemiological evidence has further shown that regardless of symptoms, as a state of dysbiosis, BV is associated with adverse reproductive health outcomes, including adverse pregnancy outcomes such as preterm birth, and acquisition of sexually transmitted infections including HIV and HPV [9–11]. Recently, evidence is also mounting that BV might negatively impact human reproduction [12–14].

Understanding community ecology and community dynamics of the vaginal microbiome may therefore offer an avenue to modulate and protect the vaginal microbiota, which, provided feasible, may have a considerable impact on preventing disease in women, their partners, and their offspring.

In the human vagina, the epithelium enriches the lumen with glycogen [15], as the primary carbon source to the resident lactobacilli, free vaginal glycogen content strongly correlating with *Lactobacillus* dominance [16–18]. Vaginal glycogen enrichment is considered a primary driving force that allows the human vagina to exert strong selective pressure in allowing bacteria to reside in this microenvironment [18–20]. In the Human Microbiome Project it was indeed shown that of all body sites accounted for, the vagina shows the lowest species diversity at the currently handled phylogenetic resolution level [21]. Merely four species belonging to the large and phylogenetically diverse *Lactobacillus* genus are found to dominate the vaginal microbiome in majority of women, i.e. *L*. *crispatus*, *L*. *iners*, *L*. *jensenii*, and *L*. *gasseri*, most often without displaying co-dominance and further accompanied by a number of unrelated species mostly occurring at low abundances [1]. Vaginal lactobacilli are incapable of glycogen fermentation however [22], glycogen breakdown presumably involving human α-amylase [23,24] and at least one other, as yet uncharacterized vaginal enzyme [25], generating the glucose oligomers maltose, maltotriose, and maltotetraose as the direct carbon sources to the resident community. Under the physiological high carbon dioxide and low oxygen levels that prevail in the vaginal environment, the vaginal *Lactobacillus*-dominated microbiota in turn produce high levels of lactic acid (mean = 1.0% w/v, SD 0.2) in a racemic mixture of D- and L-isomers, thereby acidifying the vagina to an average pH of 3.5 (standard deviation (SD) 0.3, range 2.8–4.2) [26]. *L*. *iners* differs from the other vaginal key species, as it lacks the gene coding for D-lactate dehydrogenase, yet is capable of converting glucose to pyruvate and pyruvate into L-lactic acid [27].

Lactic acid is a lipid-soluble membrane-permeant carboxylic acid that in accordance with its pKa value of 3.9, is predominantly present in the acidic vaginal environment (average pH 3.5, SD 0.3, range 2.8–4.2) as protonated lactic acid and to a much lesser extent as the unprotonated lactate [11,26,28,29]. It is generally assumed that by creating an acidic environment through lactic acid production, vaginal lactobacilli preserve their own dominance in this habitat, supported by *Lactobacillus* growth characteristics *in vitro* [30]. Although difficult to prove this mechanistic view *in vivo*, free glycogen concentration is significantly positively associated with low pH [16], further corroborating niche construction dynamics as set forth.

Lactic acid-associated vaginal acidity is also considered a primary physicochemical barrier to colonisation by invading pathogens, as documented for instance for HIV-1 [29] and *Chlamydia trachomatis* [31]. In addition, lactic acid as such, has recently emerged as a potent innate immune factor displaying a wide array of antimicrobial and immunomodulatory effects, not explained by its acidifying effect [32]. O’Hanlon *et al* documented in a pioneering *in vitro* assay that while acidity alone modestly reduced the viability of seventeen BV-associated key bacteria, lactic acid at physiological concentrations and at physiological pH, also exerted strong direct microbicidal activity against all BV-associated bacteria included [28]. Similarly, Aldunate *et al* showed modest reductions of HIV-1 infectivity resulting from acidification, yet potent virucidal activity against HIV-1 by L-lactic acid at physiological concentrations at low pH [29]. Evidence is further building that lactic acid displays a wide array of immunomodulatory effects, further enhancing the vaginal mucosal innate and adaptive response to pathogen challenge [11,32]. Noteworthy, Witkin *et al* recently revealed that the key vaginal *Lactobacillus* species differentially express the D- and L-lactate isomers, and pointed at the putative importance of the L/D-lactic acid ratio to vaginal immune homeostasis [32,33], an issue that warrants further scrutiny.

From these recent insights, several putative prebiotic strategies to long-term protection of the vaginal microbiome emerge, including vaginal replenishing with glycogen and glucose oligomers. We hypothesize that intravaginal lactic acid administration may also provide an avenue to the sustainment of an advantageous vaginal microbiome by creating an environment that enhances the recruitment of lactobacilli, while counteracting BV-associated bacteria. Lactic acid gels are commercially available and haven proven particularly useful in our experience, yet also have several disadvantages, such as frequent administration possibly leading to poor compliance, and leakage, a side-effect that is particularly undesirable in women with a history of vaginal discharge. We therefore aimed to develop an intravaginal device that would be easy to insert and remove, while providing sustained DL-lactic acid release into the vaginal lumen.

## Methods

### Vaginal ring manufacture

Ethylene vinyl acetate (EVA) was chosen as the primary copolymer, considering its established use for intravaginal delivery systems. A pure EVA matrix did not allow us however to load a sufficient amount of lactic acid and to obtain the desired release profile. Various polymer blends were subsequently developed through hot-melt extrusion and explored with regard to the overall study purpose. The final prototype selected for clinical study is a vaginal ring matrix system (external diameter 54 mm, thickness 4mm) consisting of a mixture of EVA 28 (28% w/w vinyl acetate, 72% w/w ethylene) (Celanese, EVA Performance Polymers, Edmonton, US) and the methacrylic acid-methyl methacrylate copolymer (1:1) Eudragit L100 (Evonik Industries AG, Darmstadt, Germany) in a ratio of 80.5/19.5 (% w/w), loaded with 150 mg DL-lactic acid (Fagron, Waregem, Belgium) with an L/D-lactic acid ratio of 1:1.

EVA 28 and Eudragit L100 were mixed with the lactic acid in a planetary mixer. When homogeneously blended, the mixture was extruded using a co-rotating twin screw extruder (Haake MiniMHE, Thermo Electron, Karlsruhe GmbH, Germany) at 90°C and the extrudate was subsequently injection moulded (Haake Mini Jet, Thermo Electron, Karlsruhe GmbH, Germany) into a ring mould at 800 bar and room temperature with a post pressure of 400 bar. Finally the ring was removed from the mould. The rings were GMP manufactured and individually packed in Alu/Alu sealed bags.

### In vitro release assessment

Basic screening of *in vitro* lactic acid release without accounting for vaginal buffering capacity was performed by placing ring segments of approximately 4.5 cm in a sterile recipient with 5mL demineralized water and incubated at 37°C for 10 days. The medium was replaced every 24 hours and the pH was measured daily for 10 consecutive days prior to replacement of the medium. With the final investigational product, pH of the medium ranged between 2.9 and 3.7 during this period.

### Preclinical safety assessment

Preclinical safety assessment was performed by use of the Slug Mucosal Irritation (SMI), a non-vertebrate assay to evaluate mucosal irritation that we have developed [34] and previously used for various vaginal products, also in comparison with the rabbit vaginal irritation test [35–37]. Briefly, both the matrix system with (formulation A) and without racemic lactic acid (formulation B) were tested with the SMI. Drum dried waxy maize (DDWM) starch treated slugs were used as negative controls (NC), while slugs treated with DDWM/sodium lauryl sulphate (DDWM/SLS, 80:20) were used as positive controls (PC). The slugs were placed daily on 20 mg test substance (formulation A, formulation B, NC, and PC respectively) in a Petri dish for 30 minutes a day for 5 consecutive days. For each test substance five slugs were used.

The amount of mucus produced by the slugs during each contact period was measured by weighing the Petri dish with the test substance before and after the 30-minute contact period. Mucus production (MP) was expressed as percentage (w/w) of the body weight of the index slug. After each 30-minute contact period, the slugs were placed on a membrane filter (cellulose acetate 0.45 μm, Sartorius, Germany) moistened with 2 mL PBS buffer solution in a Petri dish until the next contact period. The irritation potency of the formulations is estimated by the total mucus production during the 5-day testing period (sum of proportional mucus mass produced during each 30-minute contact period). The assay distinguished between four irritation categories: no irritation (total MP ≤ 7%), mild (7% > total MP ≤ 12%), moderate (12% > total MP ≤ 20%) and severe irritation (total MP > 20%).

### Lactic acid assay

Lactic acid load of the intravaginal ring was determined using a validated high performance liquid chromatography (HPLC) method. Briefly, following dissolution of the ring in chloroform, lactic acid was extracted with an aqueous pH 7 buffer. The HPLC system consisted of a Prevail Organic Acid column (250 x 4.6 mm; 5 μm) and a guard column. The mobile phase consisted of a 25 mM potassium platform buffer (pH 2.5). The pump flow was set at 1 mL per minute. The HPLC system consisted of a water Alliance 2695 separation module and a Photodiode Array detector and the detection wavelength was 220 nm. The eventual prototype was developed at Ghent University. However, for production of the intravaginal delivery system under GMP conditions intended for clinical investigation, license was given to a private, university spin-off company, SEPS Pharma (currently AmatsiSEPS), for manufacture of the investigational product for study purposes.

### Mechanical testing

Maximum load, tensile strain at maximum load and Young’s modulus ware determined just after manufacturing and after lactic acid release using a tensile strength machine (Ametek LF Plus, Lloyd Instruments, Heaceham, UK) at room temperature and 65% relative humidity.

### Microbiological analysis

Incubation of the rings before sealing revealed no growth on Tryptone Soya Agar and no presence of *Staphylococcus aureus*, *Pseudomonas aeruginosa* nor *Candida albicans*.

### Subject recruitment

For the first-in-human phase I clinical trial, six healthy nulliparous premenopausal volunteering women (median age 25 years, range 23 to 32 years) were recruited by the DRUG Research Unit Gent, a phase 0-I-II clinical trial unit (see study flow and ethical considerations below). Screening examinations included a standardized questionnaire on medical and gynaecologic history. Prior to enrolment all subjects screened negative on pregnancy testing. Volunteers were also subjected to a thorough gynaecological assessment to ascertain the absence of vulvar, vaginal, and cervical anomalies and the absence of pelvic floor dysfunction.

None of the subjects had a history of uterine, vaginal, or vulvar surgery, nor a history of vaginal infectious disease, except for occasional *Candida* vulvovaginitis. None of the subjects declared to have ever used intravaginal products or devices except for tampon use and occasional use of a topic antifungal drug preparation. A vaginal swab specimen (Eswab™, Copan, Brescia, Italy) was obtained from the midportion of the vaginal vault and served for Nugent scoring [38] and culture. All subjects eventually included showed a *Lactobacillus*-dominated microbiota with Nugent scores between 1 and 3, while yeast culture was negative in all subjects. The presence of *Chlamydia trachomatis* was ruled out by use of an endocervical swab specimen (UTM™, Brescia, Copan, Italy).

All volunteering women included used a combined oral contraceptive (COC) pill upon inclusion and were instructed to continue their COC for the duration of the study to avoid withdrawal bleeding. Study subjects were further asked to abstain from penetrative sexual contact and to avoid the use of any vaginal product or device for at least 72 hours and 7 days respectively, before insertion of the vaginal ring. Concomitant drug use was not allowed.

### Ethical considerations and study flow

All study subjects gave their oral and written informed consent to participate in the study. Subjects were compensated for their time and expenses related to participation in the study. The study was conducted in accordance with the tenets of the Declaration of Helsinki and current ICH-GCP guidelines. The principal investigator (PI) holds a GCP certificate. The study has been registered with the European Clinical Trials Database (EudraCT 2013-001120-19) and with the U.S. National Institutes of Health ClinicalTrials.gov registry (identifier NCT02314429).

Initial ethical approval was obtained from the Ghent University Institutional Review Board (IRB) on April 4 2013 (reference EC2013/088). The Federal Agency for Medicines and Health Products (FAGG) labelled the investigational product as a study drug (rather than a device) and requested additional technical and preclinical data, owing to which approval for safety evaluation was granted one year later, on April 25 2014, endorsed by the IRB on April 29 2014. Specifically, the FAGG gave conditional approval for a staged approach, allowing us to conduct the study in two healthy volunteers, further study conduct depending on intermediate reporting.

After revision of the original files for which approval was granted, a final protocol (S1–S3 Files) and a final informed consent form (S4 File) were submitted to the IRB as an amendment to the original files, which the IRB approved on July 15 2014. The final protocol and the final informed consent form are available as Supplementary Materials (S1 and S4 Files, respectively) accompanying this paper.

**Fig 1:**
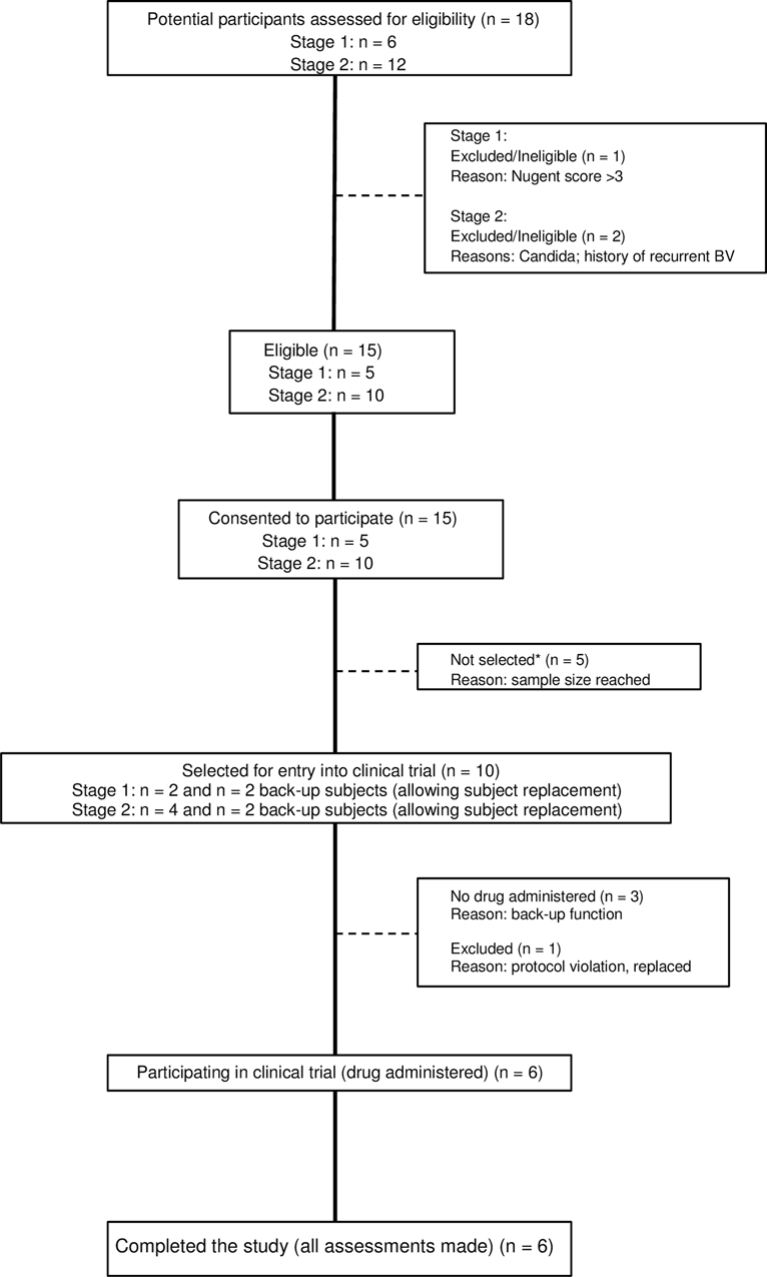
TREND study flowchart of the phase I trial. Legend: *patients were selected based on chronological order of enrolment. To ascertain that the study could take place as planned, an excess number of eligible study participants were recruited. Similarly, at each stage two back-up study subjects were standby to allow for rapid replacement of subjects, as proved necessary in one case, who violated the prohibitions explained in the informed consent form.

The study flow is presented in detail as a TREND [39] flowchart (Fig 1).

Following final, conditional approval DRUG Research Unit Gent started recruiting volunteers. DRUG is an accredited phase 0-I-II clinical trial unit that was positively evaluated by the US Food and Drug Administration. DRUG maintains a database with more than 2,000 volunteers willing to participate in clinical trials, from which eligible women were invited through e-mail until six candidate study participants were recruited from mid-July to mid-August 2014.

Six candidate study participants gave their informed consent on August 21 2014, following general health screening by a clinical trial physician of DRUG and a gynaecological screening exam by the principal investigator (PI). Of these, two subjects were withheld to enter the first stage of the trial, while another two subjects served as back-up study subjects allowing replacement of subjects when needed.

The actual trial with insertion of the investigational product and follow-up as detailed below, took place within the clinical facility of the DRUG Research Unit Gent at Ghent University Hospital from September 8 to September 15 2014. An intermediate report based on the study data obtained during this stage was send to the Federal Agency for Medicines and Health Products (FAGG) and to Ghent University Institutional Review Board (IRB) September 19 2014. The FAGG and the IRB subsequently granted their approval for the further study conduct on September 22 and 23 2014, respectively.

Accordingly, from late September until the end of October 2014, DRUG re-invited eligible women through a mailing, until twelve candidate study participants were recruited. Eventually, six candidate study participants gave their informed consent on November 17 and another six on November 20 2014, following general health screening by a clinical trial physician of DRUG and a gynaecological screening exam by the PI. Of these, four subjects were withheld to enter the second stage of the trial, while another four subjects served as back-up study subjects allowing replacement of subjects when needed. One subject was replaced by a back-up study subject immediately before investigational drug administration, as she disclosed sexual intercourse less than 12 hours before the planned intravaginal device insertion. Subject AN006 reported a cough during the study, was subsequently diagnosed with bronchitis, and was therefore followed until cure was established until mid-January 2015.

On February 5 2015 we submitted our final report along with the adverse event (AE) list (S5 File) to the Federal Agency for Medicines and Health Products (FAGG) and to Ghent University Institutional Review Board (IRB). On September 29 2015 Bimetra Clinical Research Centre Ghent conducted a monitoring assessment of all study files, following which a Declaration of the End of Trial Form was submitted to the IRB and the FAGG.

### Study procedures

The investigational device, as specified above, an intravaginal polymer ring loaded with 150 mg DL-lactic acid in a 1:1 racemic mixture, was inserted into the upper vagina by the PI, after the volunteer assumed a classic dorsal lithotomy position, left in place for precisely 7days, and removed by the PI at the occasion of the final safety assessment.

Colposcopic evaluation of the cervix and the vagina was performed by use of a stationary colposcope (Carl Zeiss, Oberkochen, Germany) at magnification x10, after placement of a disposable, sterile, transparent plastic speculum without lubrication. Colposcopy findings were recorded according to the WHO/CONRAD guidelines for the evaluation of vaginal products [40]. Colposcopic evaluations were performed one hour following insertion of the device, respectively after two, four, eight and 24 hours, and again after seven completed days.

Though no effect on vaginal pH was aimed for, nor expected, we wanted to ascertain that the vaginal pH would not drop below the physiological range. Accordingly, vaginal pH was assessed by use of a pH-Fix indicator strip (Machery-Nagel, Düren, Germany) after placement of disposable, sterile, transparent plastic speculum without lubrication, allowing to monitor the pH between a range of 3.1 to 8.3 in 0.4 increments. Vaginal pH measurements were performed each 30 minutes during the first four hours following placement of the device, each hour for the following four hours, and again after seven completed days.

On each occasion of an investigational act, naked eye inspection of the vulva, vagina, and cervix was also performed. In between these investigations, the volunteer was allowed to walk around during the first day of the study and to resume normal daily activities during the further conduct of the study.

### Adverse event reporting

During the first 8 hours after insertion of the vaginal ring, volunteers were not allowed to leave the clinical trial unit, with the PI and two clinical trial physicians in place. Volunteers were asked to spontaneously report any potential adverse event (AE), but were also actively asked for AEs before each investigational act. In case an AE was reported that did not prompt any action during this phase, follow-up was secured through telephone contacts during the further course of the study. Volunteers were also asked to record any potential AE in a diary during the entire study course. The final AE list is available through the Supplementary materials (S5 File) accompanying this paper.

## Results

### Intravaginal ring characteristics

Based on HPLC analysis, the mean lactic acid load of the intravaginal ring following manufacture varied between 90 and 110% of the label claim (150 mg).

Incubation of the intravaginal ring (n = 3) in demineralized water at 37°C for 24 hours showed release of on average 9.20 mg (SD 0.52), corresponding to an estimated 6.13% (SD 0.35) of the overall lactic acid load.

The maximum load after manufacturing and after leaching of lactic acid was 39.6 N (SD 1.6) and 43 N (SD 2.3), respectively. The tensile strain at maximum load was 401.2 and 373.2 after manufacturing and leaching of lactic acid, respectively. The Young’s modulus was 115.4 Pa (SD 23.1) and 88.3 Pa (SD 7.3) after manufacturing and leaching, respectively.

### Preclinical safety assessment

Slugs exposed to the intravaginal ring matrix with and without DL-lactic acid (1:1) produced a minimal amount of mucus during contact, with a total MP of 3.3% w/w of body weight (SD 0.9) and 3.8% w/w of body weight (SD 1.6) on average for slugs treated with formulation A and B, respectively, and hence the investigational product produced no irritation in the SMI test (total MP ≤ 7%). This is comparable with the total amount of mucus produced by the negative control slugs (5.6% w/w of body weight, SD 1.3). Positive control slugs on the other hand showed a high total MP (23.7% w/w of body weight, SD 3.6) indicating severe irritation (total MP > 20%).

### Safety and tolerability assessment in healthy volunteers

All volunteering women enrolled (n = 6) completed the study. Naked eye inspection did not reveal any vulvar lesions during the 7-day study course. Similarly, colposcopic examination of the vagina and the cervix did not show any sign of epithelial disruption, oedema, colour change, or subepithelial bleeding as the vaginal ring was in place. In one subject, at some point in time during the initial safety assessment phase, insertion of the speculum provoked a transient bleeding at the cervical ectropion site, however without hindering colposcopic assessment. Vaginal pH at initial assessment ranged between 3.1 and 4.3 and remained largely constant throughout the initial 8 hour safety monitoring phase, except in the subject with cervical ectropion bleeding who maintained a pH of 4.7. At the final safety assessment of the 7-day study course, vaginal pH in all subjects ranged between 3.5 and 4.3.

A total of 11 adverse events (AEs) were reported by four subjects (S5 File). All AEs were considered to be unrelated to the investigational device and hence required no action with regard to the conduct of the study. Seven AEs did concern the pelvis. In subject AN001 a breakthrough bleeding following continuous COC use occurred on day 7, first noticed two hours before removal of the ring. In subject AN002 mild bleeding of the cervical ectropion was noticed following the first speculum examination, which appeared to have completely resolved during the colposcopic assessment 8 hours post-insertion. The latter subject also reported a mild pressure sensation in the lower abdomen 10 hours after placement of the ring, which she ascribed to the repeated gynaecological examinations during that day. Subject AN005 reported two transient episodes of discomfort over the posterior vaginal vestibular area on day 1, which she attributed to the repeated speculum insertions. Finally, subject AN006 reported a vaginal foreign body sensation 12 hours after insertion of the device, which she also ascribed to the investigational procedures. Hence, except for the breakthrough bleeding in subject AN001, all pelvic AEs occurred during the first day of the study involving twelve speculum examinations during the first 8 hours and were considered to be procedure-related. Consequently, no such pelvic AEs were reported during the further study course.

At the end of the study, all study subjects were briefly asked for their experience with participating in the study and the acceptability of the intravaginal ring. All subjects reported mild to moderate vaginal discomfort associated with the repeated safety assessments on day 1. Similarly, all subjects confirmed not to have experienced any discomfort during the further study course with the vaginal ring in place, which they considered highly acceptable.

### Lactic acid release

HPLC analysis performed immediately following clinical testing revealed that a mean of 37.1 mg (SD 0.9) DL-lactic acid was released from the vaginal ring over the 7-day study course, indicating that the vaginal ring load might enrich the vaginal environment with DL-lactic acid for a considerably longer period then was considered in the present study.

## Discussion

We developed an intravaginal device ensuring continuous slow release of DL-lactic acid into the vaginal environment from the hypothesis that sustained lactic acid enrichment will enhance the recruitment of lactobacilli, while counteracting bacterial vaginosis-associated bacteria. The final prototype selected is a vaginal ring matrix system consisting of a mixture of ethylene vinyl acetate and a methacrylic acid-methyl methacrylate copolymer loaded with 150 mg DL-lactic acid with an L/D-lactic acid ratio of 1:1. DL-lactic acid release from the intravaginal ring during a 7-day period *in vivo* was estimated through HPLC analysis at 37.1 mg (SD 0.9). While we did not assess DL-lactic acid release *in vivo* at shorter durations, and hence are unable to document the release profile in detail, the preclinical *in vitro* assessment did not show a significant burst release with an estimated 6.13% (SD 0.35) of the overall lactic acid load released over the first 24 hours. Indirect monitoring of lactic acid release in an aqueous medium through pH measurement for 10 consecutive days was also consistent with continuous slow release during this period. No adverse effects were observed during preclinical testing with the slug mucosal irritation assay, nor in a phase I clinical trial with 6 healthy volunteers.

Novel approaches in modulating and protecting the vaginal microbiota are particularly appealing for a number of reasons.

First, such approaches might provide health care providers with an acceptable means in treating patients with recurrent bacterial vaginosis (BV), a particularly prevalent and frustrating condition negatively impacting on women’s quality of life. Historically, and particularly due to the emphasis on *Gardnerella vaginalis* as the presumed causative agent since the 1950s, BV has been considered as an infectious disease which has led to a number of clinical trials with a wide array of antibiotics [41]. Two hallmark studies, with metronidazole [42] and clindamycin [43], respectively, eventually set the scene for current treatment guidelines [41]. Basically, antibiotic treatment of BV is understood to clear the vaginal niche from its highly diverse polymicrobial community, rapidly leading to symptomatic relief, while allowing lactobacilli to re-occupy this vacated niche. High recurrence rates are however observed with standard antibiotic treatment [5], even after a prolonged treatment course of 16 weeks [44].

From human microbiome research in recent years, the notion of a ‘bacterial community as a pathogen’ emerged as a novel concept in microbial pathogenesis, in which a conserved microbial assemblage, rather than any one specific member, contributes to disease [45]. Accordingly, when considering the pathogenicity of the microbiome, it might be better to focus on bacterial community ecology and dynamics [46]. As a community state, BV is actually a rather stable community disturbance presumably also due to biofilm formation [47] and community stability should therefore not be misconstrued with healthy or advantageous microbiota [48]. Hence, novel approaches as proposed in the current study, that alter the vaginal niche through a prebiotic approach enhancing vaginal re-colonisation with lactobacilli are therefore not confined to recurrent symptomatic BV, but also have a tremendous potential in preventing bacterial vaginosis-associated disease in women, their partners, and their offspring.

A particularly interesting alternative approach is the administration of probiotic lactobacilli, subsequent evidence-based assessments [49–52] all precautiously pointing at positive proof-of-concept in the absence of sufficiently strong evidence at present in recommending probiotics in the treatment of recurrent BV. Recent whole genome sequencing approaches now allow for unprecedented comprehensive screening of probiotic functionality of selected vaginal key *Lactobacillus* species such as selected *L*. *crispatus* strains [53–56], than was previously achieved with cultured isolates, and therefore provide another promising avenue for the development of vaginal probiotics. Finally, synbiotic approaches, combining probiotics and prebiotics, are another therapeutic and prophylactic perspective in modulating and protecting the vaginal microbiota

The prebiotic approach has been previously explored, especially with regard to lactic acid. Holst and Brandberg reported on a small, non-controlled trial in 10 pregnant women with incident BV who administered a vaginal lactic acid gel intermittently for 8 weeks [57]. The authors reported that all women reverted to a lactobacilli-dominated after 2 days of treatment, 8 women maintaining clinical and microbiological cure at the end of the trial [57]. Andersch *et al*, recruited 42 women with recurrent BV (3 or more documented episodes during a 12-month period prior to enrolment) and initially treated all women with vaginal lactic acid gel, administered for 7 consecutive days at night [58]. Study participants were subsequently randomized in two arms in a double-blinded fashion and instructed to administer the vaginal lactic acid gel or an identical placebo for 3 consecutive days after menstruation or withdrawal bleeding for 6 months. At the end of the trial, clinical cure was documented for 88.2% (15/17) of women in the lactic acid group versus 10% in the placebo group (1/10), and microbiological cure was found with 87.5% (14/16) of patients with the active drug versus 10% in the placebo group (1/10) [58]. Even when accounting for substantial drop-out in this study, continued intermittent lactic acid treatment therefore appears promising in long-term treatment of BV. It should be acknowledged however, that the investigational product in the latter study actually consisted of lactic acid (5% w/v) as well as glycogen (0.1% w/w). Albeit not addressed by the authors, glycogen may have contributed to the observed effects. In a large randomized, double-blinded, three-arm comparison of a single course of oral metronidazole for 7 days, lactic acid (100 mg) vaginal suppositories for 7 days, and placebo treatment for the treatment of incident BV, Boeke *et al* found that cure rates with lactic acid suppositories were not different from placebo and significantly less effective than metronidazole at 4 weeks and 3 months, respectively [59]. Though the latter study was rigorously designed, the authors did not provide details on the lactic acid suppository manufacturing, which may be critical to lactic acid release, which was also not reported. Finally, in an open-labeled study, Decena *et al*, found vaginal lactic acid gel, oral metronidazole, and a combination of those to be equally effective in short-term cure of incident BV [60]. Hence, while disparate results have been obtained in the treatment of incident BV, one study [58] does provide preliminary evidence on the potential of lactic acid (though combined with glycogen) in modulating and protecting the vaginal microbiota for a prolonged period of time, and therefore warrants further scrutiny.

A major advantage of the polymer device presented here involves the easiness to insert and remove, while avoiding administration of semisolid vaginal formulations such as suppositories and gels, which are of concern because of several reasons. An obvious disadvantage of such formulations is the frequent administration, limited residence time, and significant leakage from the vagina [61]. Secondly, the safety of such extremely high doses of lactic acid, relative to physiological concentrations, has not been established. Thirdly, while vaginally applied products have been a general issue of concern since the nonoxynol-9 story [62–64], recent systems biology approaches have further increased such concern with regard to microbicides and even to vaginally applied excipients in microbicide formulations, transcriptomic studies revealing significant mucosal and immune toxicity with commercial and investigational formulations previously considered safe [65–67]. Intravaginal devices such as intravaginal rings allow to circumvent such toxicity by slowly releasing only the desired drug, which in this case is even a primary physiological constituent of this niche.

While we have therefore shown at present the feasability of enriching the vaginal environment with DL-lactic acid with a prototype intravaginal ring, further development of the present invention is warranted. First, the ethylene vinyl acetate/methacrylic acid-methyl methacrylate matrix we used showed significantly less rigidity compared to presently available intravaginal ring systems. While this may add to patient comfort, it also increases the risk for expulsion of the intravaginal ring [68]. Second, while we aimed to mimic physiological DL-lactic acid concentrations present with lactobacilli-dominated microbiota, we are uncertain at present whether the release obtained will be sufficient to obtain the desired prebiotic effect. Third, as mentioned above, it was recently suggested that the L/D-lactic acid ratio may also be of importance to vaginal immune homeostasis [32,33], and therefore the L/D-lactic acid ratio with our system might be optimized. Here, we handled an L/D-lactic acid ratio of 1 which is close to what is observed with *L*. *crispatus* and significantly lower than the L/D-lactic acid ratio observed with a *L*. *iners*-dominated microbiome, suggesting that an L/D-lactic acid ratio of 1 might actually be quite favourable [69].

## Supporting Information

S1 File Study protocol (English version). (DOCX)

S2 File Study protocol–Original version (Dutch). (DOC)

S3 File Protocol clarification Letter (Dutch). (DOC)

S4 File Informed Consent Form (Dutch). (DOCX)

S5 File Adverse Events List (English). (PDF)

S6 File TREND Checklist. (PDF)
