# Supplementary material for: Rationale and Safety Assessment of a Novel Intravaginal Drug-Delivery System with Sustained DL-Lactic Acid Release, Intended for Long-Term Protection of the Vaginal Microbiome
Source: PLoS One. 2016 Apr 19;11(4):e0153441. doi: 10.1371/journal.pone.0153441 (PMC4836750; doi:10.1371/journal.pone.0153441)
Supplement: S1 File — (DOCX) [file pone.0153441.s001.docx]

Part I : Trial related part of the protocol

# Title of the trial

Phase I trial evaluating the safety of a lactic acid releasing vaginal ring for the prophylaxis of bacterial vaginosis

# Trial number

Protocol: LACRING01

EudraCT number: 2013-001120-19

# Objective of the study

Phase I trial to evaluate the safety of an intravaginal polymer ring releasing a racemic mixture of lactic acid; in particular, this study aims to ascertain the absence of toxic effects on the cervicovaginal epithelium in healthy volunteers (n=6).

# General information

## Investigator(s)

**Prof. dr. Hans Verstraelen**

Vrouwenkliniek

Vakgroep Uro-Gynaecologie

Universiteit Gent

De Pintelaan 185

B-9000 Gent

T. 09/332 22 23

f. 09/332 38 31

e-mail: hans.verstraelen@ugent.be

**Prof. dr. Jean-Paul remon**

Vakgroep Geneesmiddelenleer

FFW, Universiteit Gent

Harelbekestraat 72

B-9000 Gent

t. 09 264 80 54

f. 09 222 82 36

e-mail: jeanpaul.remon@ugent.be

**Prof. dr. Chris Vervaet**

Vakgroep Geneesmiddelenleer

FFW, Universiteit Gent

Harelbekestraat 72

B-9000 Gent

t. 09 264 80 54

f. 09 222 82 36

e-mail: chris.vervaet@ugent.be

## Sponsor

Ghent University

## Departments/laboratories involved in the study

Department of Obstetrics & Gynaecology, Faculty of Medicine and Health Sciences, Ghent University, Ghent, Belgium

Department of Pharmaceutics, Laboratory of Pharmaceutical Technology, Faculty of Pharmaceutical Sciences, Ghent University, Ghent, Belgium

# Introduction

In the 1980’s, several clinical trials were conducted with vaginal lactic acid gels [Andersch et al, 1986; Andersch et al, 1990; Holst & Brandberg, 1990], and more recently repeated in comparison to metronidazole [Decena et al, 2006], all of these studies aiming at long-term prophylaxis of bacterial vaginosis.

Lactic acid gels have several disadvantages however: (1) frequent administration possibly leading to poor compliance, (2) leakage, and (3) potential toxicity of the lactic acid concentrations used on the vaginal epithelium.

The intravaginal ring is a continuous *slow release* system for the vaginal delivery of lactic acid, as described in patent application P/2011-035-intravaginal delivery system (EP 111 74 103.9), without the aforementioned disadvantages, and may stay in place for a prolonged period of time.

*Andersch B, Forssman L, Lincoln K, Torstensson P. Treatment of bacterial vaginosis with an acid cream: a comparison between the effect of lactate-gel and metronidazole. Gynecol Obstet Invest. 1986;21(1):19-25.*

*Andersch B, Lindell D, Dahlén I, Brandberg A. Bacterial vaginosis and the effect of intermittent prophylactic treatment with an acid lactate gel. Gynecol Obstet Invest. 1990;30(2):114-9.*

*Holst E, Brandberg A. Treatment of bacterial vaginosis in pregnancy with a lactate gel. Scand J Infect Dis. 1990;22(5):625-6.*

*Decena DC, Co JT, Manalastas RM Jr, Palaypayon EP, Padolina CS, Sison JM, Dancel LA, Lelis MA. Metronidazole with Lactacyd vaginal gel in bacterial vaginosis. J Obstet Gynaecol Res. 2006 Apr;32(2):243-51*

# The present study

## Study design

First-in-human phase I clinical trial, in which in 6 volunteering women with normal vaginal microbiota, a intravaginal lactic acid (7.5%) releasing ring (as described in European Patent EP 111 74 103.9), manufactured under GMP will be inserted in healthy volunteering women and left in place for 7 days.

The study will be conducted in a staged approach. In a first stage, the intravaginal ring will be administered to 2 healthy volunteers. The second stage with another 4 healthy volunteers, can only be completed provided the IRB and the FAGG give secondary approval to the conduct of the study following intermediate reporting after the first stage with 2 healthy volunteers.

## (Medical Device)- Drug

### Composition and dosing

- Ethylene vinyl acetate copolymer EVA 28: 72.5%
- Methacrylic acid - methyl methacrylate copolymer Eudragit L100: 20.0%
- DL-lactic acid (L/D ratio 1:1): 7.5%

For one ring this equals

- EVA 28: 1.45 g

- Eudragit L: 0.40 g

- DL-lactic acid: 0.15 g

### Producer

SEPS Pharma

Technologiepark 4, 9052 Gent, Belgium

### Packaging

GMP manufactured rings are individually packed in Alu/Alu sealed bags.

### Administration way

intravaginal

### Storage conditions

Stored at room temperature.

### Known side effects of the medical device.

none

### Accountability

Ghent University

## The subjects

### Number of subjects

6 healthy volunteering women

### Inclusion criteria

- women aged 18 to 45 years (including limits)
- good general physical and mental health
- presence of normal (lactobacilli-dominated) vaginal microbiota
- using oral contraception and/or prepared to take oral contraception for the purpose of the study in a such manner that no uterine bleeding is expected during the study course (screening visit and study days D1 to D8)

### Exclusion criteria

- known disease, i.e. major systemic disease such as Crohn’s disease, auto-immune disorders, diabetes mellitus, etc. Minor disease not expected to impact the study results (e.g. hay fever) will not lead to exclusion
- pregnancy
- lactation
- menopause
- use of vaginal drugs or devices 7 days or less before study onset
- use of antibiotics 7 days or less before study visit 2

### Replacement of subjects

If a study subject cannot complete the study for any reason, the subject will be replaced by another study subject, until the sample size is reached.

### Restrictions and prohibitions for the subjects

Subjects should abstain from penetrative sexual contact for at least 72 hours before study visit 2.

Use of vaginal drugs or devices should be avoided at least 7 days before study visit 2.

Continue oral contraception during the study.

### Possible advantages and risks for the subjects

Subjects will not benefit directly from the study.

No major risks are expected, though irritation of the vaginal mucosa may occur, following which the intravaginal ring will be removed.

# Procedures

## Procedures

After insertion of the vaginal ring, colposcopy with thorough inspection of the cervicovaginal surface is performed after one hour, respectively after 2 hours, 4 hours, 8 hours, 24 hours and 7 days post dose.

After insertion of the vaginal ring, vaginal pH is assessed each 30 minutes for 4 hours, and then each 60 minutes for the next 4 hours.

## Flowchart

The study will take an estimated 3 months. The time lag between the screening visit and visit 2 shall not exceed 4 weeks.

**Visit 1** (screening/inclusion)

- providing study information – informed consent – inclusion
- general health screening
- pregnancy testing
- gynaecological exam with sample collection for vaginal microbiota status and Chlamydia

**Visit 2** (day 1/D1)

- insertion ring
- after 30’, pH measurement
- after 1h, colposcopy and pH measurement
- after 1h30’, pH measurement
- after 2h, colposcopy and pH measurement
- after 2h30’, pH measurement
- after 3h, pH measurement
- after 3h30’, pH measurement
- after 4h, colposcopy and pH measurement
- after 5h, pH measurement
- after 6h, pH measurement
- after 7h, pH measurement
- after 8h, colposcopy and pH measurement

**Visit 3** (day 2/D2)

- colposcopy

**Visit 4** (day 8/D8)

- removal ring
- colposcopy

# Randomisation/blinding

none

# Prior and concomitant therapy

No use of vaginal drugs or devices from 7 days before study onset until completion of the study.

No use of local or systemic antibiotics from 7 days before study onset until completion of the study.

# Adverse event reporting

Abbreviations:

AE Adverse Event

CA Competent Authority

EC Ethics Committee

SAE Serious Adverse Event

SADE Serious Adverse Device Effect

USADE Unanticipated Serious Adverse Device Effect

A serious adverse event is one that:

a) Led to a death,

b) Led to a serious deterioration in the health of the subject that:

1) Resulted in a life-threatening illness or injury, or

2) Resulted in a permanent impairment of a body structure or a body function,

or

3) Required in-patient hospitalization or prolongation of existing hospitalization, or

4) Resulted in medical or surgical intervention to prevent life threatening illness or injury or permanent impairment to a body structure or a body function,

c) Led to fetal distress, fetal death, or a congenital abnormality or birth defect.

An untoward medical occurrence that happens in a subject or other person, is related to the investigational device, comparator, or procedure, and is serious, but is *not unanticipated* is a serious adverse device effect (SADE).

An untoward medical occurrence that happens in a subject or other person; is related to the investigational device, device procedure, or comparator; is serious; and was unanticipated is classified as an unanticipated serious adverse device effect (USADE).

Adverse events will be reported between the first use of the medical device, and the last trial related activity.

All AEs and SA(D)E’s will be recorded in the patient’s file and in the CRF. All SADE’s will be reported as described below.

All SA(D)E’s occurring during the clinical trial must be reported by the Principal Investigator within 2 working days after becoming aware of the SA(D)E to:

- The EC
- Bimetra Clinics of the University Hospital Ghent
- The producer of the investigational medical device

For the contact details, see below.

In case the investigator decides the SAE is a USADE, Bimetra Clinics will report this to the Central EC and the CA.

In case of a life-threatening USADE the entire reporting process must be completed within 7 calendar days. In case of a non-life-threatening USADE the reporting process must be completed within 15 calendar days.

The first report may be made by telephone, e-mail or facsimile (FAX).

Contact details of Bimetra Clinics:

e-mail: Bimetra.Clinics@uzgent.be

tel.: 09/332 05 00

fax: 09/332 05 20

Contact details of the National Coordinating Investigator:

Prof. dr. H. Verstraelen

e-mail: Hans.Verstraelen@ugent.be

tel.: 09/332 22 23

fax: 09/332 38 31

Contact details of the producer of the medicinal (investigational) product::

e-mail: Jody.Voorspoels@sepspharma.com

tel.: 09/261 69 01

fax: 09/261 69 20

The investigator must provide the minimal information: i.e. trial number, subject's initials and date of birth, period of intake, nature of the adverse event and investigator's attribution.

Reporting by telephone must always be confirmed by a written, more detailed report. Pregnancies occurring during clinical trials are considered immediately reportable events. They must be reported as soon as possible. The outcome of the pregnancy must also be reported.

**If the subjects are not under 24-hour supervision of the investigator or his/her staff (out-patients, volunteers), they (or their designee, if appropriate) must be provided with a "trial card" indicating the name of the investigational product, the trial number, the investigator's name and a 24-hour emergency contact number.**


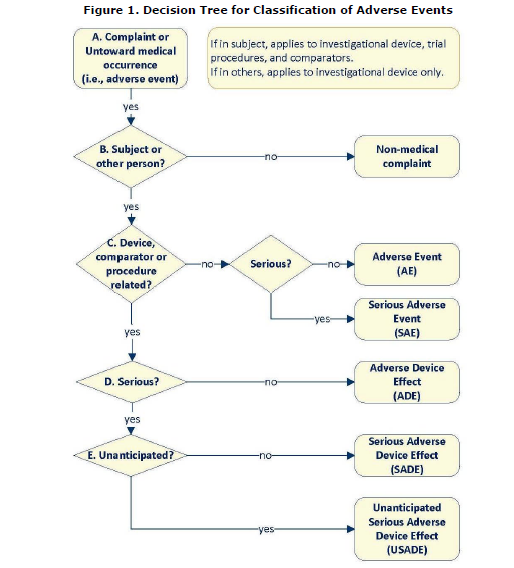


# Study analysis

## Sample size calculation

No sample size calculations were made. Six study subjects were deemed sufficient for a first-in-human approach.

## Analysis of the samples

No clinical samples will be obtained during the study following screening.

## Statistical analysis

The study will only generate descriptive data – no statistical analyses are planned.

# Quality control and quality assurance

Quality control of the CRF data will be performed by an independent clinical trial assessor from Bimetra Clinical Research Centre, not involved in the study conduct, including comparison of CRF data with source data.

# Indemnity insurance

No fault polis Ghent University.

# Publication policy

Data obtained in the study will be published in an international scientific journal, without identification data.

Part II : General part of the protocol

# Independent Ethics Committee (IEC) / Institutional Review Board (IRB)

This trial can only be undertaken after full approval of the protocol and addenda has been obtained from the IEC/IRB. This document must be dated and clearly identify the protocol, amendments (if any), the informed consent form and any applicable recruiting materials and subject compensation programs approved.

During the trial, the following documents will be sent to the IEC/IRB for their review:

- reports of adverse events as described in section 10: “Adverse Event reporting”
- all protocol amendments and revised informed consent form (if any).

Amendments should not be implemented without prior review and documented approval / favorable opinion form the IEC/IRB except when necessary to eliminate an immediate hazard to trial subjects or when the change involves only logistical or administrative aspects of the trial.

Reports on, and reviews of the trial and its progress will be submitted to the IEC/IRB by the investigator at intervals stipulated in their guidelines.

At the end of the trial, the investigator will notify the IEC/IRB about the trial completion.

# ICH/GCP guidelines

This trial will be conducted in accordance with the protocol, current ICH-GCP guidelines and applicable law(s).

Good Clinical Practice (GCP) is an international ethical and scientific quality standard for designing, conducting, recording and reporting trials that involve the participation of human subjects. Compliance with this standard provides public assurance that the rights, safety and well-being of trial subjects are protected, consistent with the principles that have their origin in the Declaration of Helsinki, and that the clinical trial data are credible.

# Subject information and informed consent

Prior to entry in the trial, the investigator must explain to potential subjects or their legal representatives the trial and the implication of participation. Subjects will be informed that their participation is voluntary and that they may withdraw consent to participate at any time. Participating subjects will be told that their records may be accessed by competent authorities and by authorized persons without violating the confidentiality of the subject, to the extent permitted by the applicable law(s) and/or regulations. By signing the Informed Consent Form (ICF), the subjects or legally acceptable representatives are authorizing such access.

After this explanation and before entry to the trial, written, dated and signed informed consent should be obtained from the subject or legally acceptable representative. The ICF should be provided in a language sufficiently understood by the subject. Subjects must be given the opportunity to ask questions.

The subject or legally acceptable representative will be given sufficient time to read the ICF and to ask additional questions. After this explanation and before entry to the trial, consent should be appropriately recorded by means of either the subject's or his/her legal representative's dated signature or the signature of an independent witness who certifies the subject's consent in writing. After having obtained the consent, a copy of the ICF must be given to the subject.

In case the subject or legally acceptable representative is unable to read, an impartial witness must attest the informed consent.

Subjects who are unable to comprehend the information provided or pediatric subjects can only be enrolled after consent of a legally acceptable representative.

# Case Report Forms

The source documents are to be completed at the time of the subject’s visit. The CRFs are to be completed within reasonable time after the subject’s visit.

The investigator must verify that all data entries in the CRFs are accurate and correct. If certain information is Not Done, Not Available or Not Applicable, the investigator must enter "N.D." or "N.AV." or "N.AP", respectively in the appropriate space.

# Direct access to source data / documents

The investigator will permit trial-related monitoring, audits, IRB/IEC review, and regulatory inspection(s), providing direct access to source data/documents.

# Data handling and record keeping

The investigator and sponsor specific essential documents will be retained for at least 20 years. At that moment, it will be judged whether it is necessary to retain them for a longer period, according to applicable regulatory or other requirement(s).

# Signature page

*Investigator:*

Name: ________________________________________________

Title: ________________________________________________

Signature: ________________________________________________

Date: ________________________________________________

*Investigator:*

Name: ________________________________________________

Title: ________________________________________________

Signature: ________________________________________________

Date: ________________________________________________
