# Supplementary material for: Rationale and Safety Assessment of a Novel Intravaginal Drug-Delivery System with Sustained DL-Lactic Acid Release, Intended for Long-Term Protection of the Vaginal Microbiome
Source: PLoS One. 2016 Apr 19;11(4):e0153441. doi: 10.1371/journal.pone.0153441 (PMC4836750; doi:10.1371/journal.pone.0153441)
Supplement: S2 File — (DOC) [file pone.0153441.s002.doc]

# Part I : Trial related part of the protocol

# Title of the trial

Studie naar de veiligheid van een lactaat vrijstellende vaginale ring in de profylaxe van bacteriële vaginose.

# Trial number

Protocol: LACRING01.

EudraCT number: 2013-001120-19

# Objective of the study

Fase I studie waarbij de veiligheid wordt nagegaan van een vaginale polymeerring die (racemisch) melkzuur vrijstelt; in het bijzonder zal worden nagegaan of de ring geen toxische effecten genereert t.h.v. het vaginale epitheel bij gezonde vrijwilligsters (n=6).

# General information

## Investigator(s)

Prof. dr. Hans Verstraelen

Vrouwenkliniek

Vakgroep: Uro-Gynaecologie

Universiteit Gent

De Pintelaan 185

B-9000 Gent

T. 09/332 22 23

f. 09/332 38 31

e-mail: hans.verstraelen@ugent.be

Prof. dr. Jean-Paul remon

Vakgroep Geneesmiddelenleer

FFW, Universiteit Gent

Harelbekestraat

B-9000 Gent

t. 09 264 80 54

f. 09 222 82 36

e-mail: jeanpaul.remon@ugent.be

Prof. dr. Chris Vervaet

Vakgroep Geneesmiddelenleer

FFW, Universiteit Gent

Harelbekestraat

B-9000 Gent

t. 09 264 80 54

f. 09 222 82 36

e-mail: chris.vervaet@ugent.be

## Sponsor

Universiteit Gent

## Departments/laboratories involved in the study

Vakgroep Uro-Gynaecologie, Faculteit Geneeskunde en Gezondheidswetenschappen, UGent

Laboratorium voor Farmaceutische Technologie, Vakgroep Geneesmiddelenleer, Faculteit Farmaceutische Wetenschappen, UGent

# Introduction

In de jaren ’80 werden reeds verscheidene klinische studies verricht met een lactaat gel (Andersch et al, 1986; Andersch et al, 1990; Holst & Brandberg, 1990], terwijl in een meer recente studie een lactaat gel werd toegepast in combinatie met metronidazole [Decena et al, 2006], telkens ter behandeling (op lange termijn) van bacteriële vaginose.

De lactaatgels hebben evenwel verscheidene nadelen: ze vereisen frequente toediening en derhalve een hoge compliantie van de patiëntes, ze geven aanleiding tot toegenomen vaginale afscheiding en tenslotte zijn lactaat gels bij de gebruikte concentraties mogelijk ook toxisch voor het vaginale epitheel. De vaginale ring waaruit lactaat kan diffunderen en dus een *slow release* systeem vertegenwoordigt voor de vaginale toediening van lactaat, beschermd onder patentaanvraag *P/2011-035-vaginal matrix ring* (*EP 111 74 103.9)* heeft niet de hierboven beschreven nadelen zoals vaginale lactaatgels en kan gedurende langere tijd ter plaatse blijven.

*Andersch B, Forssman L, Lincoln K, Torstensson P. Treatment of bacterial vaginosis with an acid cream: a comparison between the effect of lactate-gel and metronidazole. Gynecol Obstet Invest. 1986;21(1):19-25.*

*Andersch B, Lindell D, Dahlén I, Brandberg A. Bacterial vaginosis and the effect of intermittent prophylactic treatment with an acid lactate gel. Gynecol Obstet Invest. 1990;30(2):114-9.*

*Holst E, Brandberg A. Treatment of bacterial vaginosis in pregnancy with a lactate gel. Scand J Infect Dis. 1990;22(5):625-6.*

*Decena DC, Co JT, Manalastas RM Jr, Palaypayon EP, Padolina CS, Sison JM, Dancel LA, Lelis MA. Metronidazole with Lactacyd vaginal gel in bacterial vaginosis. J Obstet Gynaecol Res. 2006 Apr;32(2):243-51*

# The present study

## Study design

Fase I studie waarbij bij 6 vrijwilligsters met een normale vaginale microbiota een onder GMP condities vervaardigde lactaat (7.5%) vrijstellende vaginale ring (zoals omschreven onder European Patent referentie *EP 111 74 103.*9) in de vagina wordt ingebracht en gedurende 7 dagen ter plaatse blijft. De studie verloopt in 2 fases. In een eerste fase zal bij 2 personen de vaginale ring worden ingebracht. Een tweede fase, met 4 proefpersonen, mag slechts worden uitgevoerd na goedkeuring van Ethisch comité en FAGG gebaseerd op een rapport met eventuele veiligheidsrisico’s na het doorlopen van de eerste fase van de studie. Bedoeling van dit fase I onderzoek is ongewenste effecten die desgevallend zouden optreden vroegtijdig op te sporen en desgevallend de dosis lactaat aan te passen.

## (Medical Device)- Drug

### Composition and dosing

- Ethyleenvinylacetaat copolymeer (EVA) 28: 72,5 %

- polymethacrylzuur-methylmethacrylaat copolymeer (Eudragit L): 20,0 %

- L - Melkzuur: 7,5 %

Voor 1 ring (2 gram) komt dit op:

- EVA 28: 1,45 gram

- Eudragit L: 0,40 gram

- L - Melkzuur: 0,15 gram

### Producer

SEPS Pharma,

Technologiepark 4,

9052 Gent

### Packaging

Elke lactaatring wordt apart verpakt in een Alu/Alu verpakking.

### Administration way

intravaginaal

### Storage conditions

De ringen zullen bewaard worden op kamertemperatuur.

### Known side effects of the medical device.

geen

### Accountability

Universiteit Gent.

## The subjects

### Number of subjects

6 vrijwilligsters

### Inclusion criteria

- verkeren in algemene goede mentale en fysieke gezondheid
- aanwezigheid van een normale (Lactobacillus gedomineerde) vaginale microbiota
- orale contraceptie gebruiken of bereid zijn deze op te starten in het kader van de studie + bereid zijn om orale contraceptie door te nemen tijdens de studie zodat er geen bloeding kan worden verwacht op één van de studiedagen (screening + D1-D8)
- vrouwen tussen 18-45 jaar (uitersten inclusief)

### Exclusion criteria

- gekende ziekte: hiermee worden belangrijke systeemziekten (vb ziekte van Crohn, reumatische aandoeningen, diabetes mellitus, …) bedoeld. Mineure aandoeningen waarvan niet verwacht wordt dat ze een invloed hebben op de studieresultaten (hooikoorts, …) vormen geen reden tot exclusie.
- Zwangerschap
- Vrouwen die borstvoeding geven
- Post-menopauzale vrouwen
- reeds gebruik van vaginale medicatie of device vanaf 1 week vóór visite 2 en tijdens de studie
- gebruik van antibiotica vanaf 1 week vóór visite 2 en tijdens de studie

### Replacement of subjects

Indien een proefpersoon de studie om welke reden dan ook de studie niet kan voltooien zal deze vervangen worden door een andere proefpersoon.

### Restrictions and prohibitions for the subjects

Proefpersonen mogen geen seksueel contact hebben vanaf 2 dagen vóór visite 2 van de studie en tijdens de studie. Er mogen evenmin vaginale producten gebruikt worden of vaginale spoelingen worden toegepast. De proefpersonen wordt gevraagd orale contraceptie op te starten, zo dit nog niet reeds het geval is.

### Possible advantages and risks for the subjects

Er is geen onmiddellijk voordeel voor de proefpersonen. Er zijn wellicht geen nadelen, tenzij mogelijks lokale irritatie t.h.v. het vaginale epitheel, waarna de studiering onmiddellijk zal verwijderd worden.

# Procedures

## Procedures

- Plaatsing vaginale lactaatring; vaginale pH meting met pH-strip om de 30 minuten gedurende de eerste 4 uren na het plaatsen van de ring en om het uur in daaropvolgende 4 uren
- colposcopie ter evaluatie van lokale neveneffecten 1 uur, resp. 2 uren, 4 uren, 8 uren, 24 uren en 8 dagen na insertie van de ring

## Flowchart

De studie zal ongeveer 3 maanden duren. De tijd tussen visite 1 en 2 mag maximaal 4 weken bedragen.

**Visit 1** (screening/inclusie)

- informatie – informed consent – inclusie
- algemene screening
- zwangerschapstest
- afname vaginaal uitstrijkje voor analyse

**Visit 2** (dag 1)

- plaatsen ring
- na 30’, meting pH
- na 1h, colposcopie en meting pH
- na 1h30’, meting pH
- na 2h, colposcopie en meting pH
- na 2h30’, meting pH
- na 3h, meting pH
- na 3h30’, meting pH
- na 4h, colposcopie en meting pH
- na 5h, meting pH
- na 6h, meting pH
- na 7h, meting pH
- na 8h, colposcopie en meting pH

**Visit 3** (dag 2)

- colposcopie

**Visit 4** (dag 8)

- verwijderen ring en colposcopie

# Randomisation / blinding

Er is geen randomisatie noch blindering.

# Prior and concomitant therapy

Proefpersonen mogen geen gebruik maken van intravaginale geneesmiddelen gedurende de week voor en tijdens de studie.

Proefpersonen mogen geen gebruik maken van systemische of lokale antibiotica gedurende de week voor en tijdens de studie.

# Adverse event reporting

Abbreviations:

AE Adverse Event

CA Competent Authority

EC Ethics Committee

SAE Serious Adverse Event

SADE Serious Adverse Device Effect

USADE Unanticipated Serious Adverse Device Effect

A serious adverse event is one that:

a) Led to a death,

b) Led to a serious deterioration in the health of the subject that:

1) Resulted in a life-threatening illness or injury, or

2) Resulted in a permanent impairment of a body structure or a body function,

or

3) Required in-patient hospitalization or prolongation of existing hospitalization,or

4) Resulted in medical or surgical intervention to prevent life threatening illness or injury or permanent impairment to a body structure or a body function,

c) Led to fetal distress, fetal death, or a congenital abnormality or birth defect.

An untoward medical occurrence that happens in a subject or other person, is related to the investigational device, comparator, or procedure, and is serious, but is *not unanticipated* is a serious adverse device effect (SADE).

An untoward medical occurrence that happens in a subject or other person; is related to the investigational device, device procedure, or comparator; is serious; and was unanticipated is classified as an unanticipated serious adverse device effect (USADE).

Adverse events will be reported between the first use of the medical device, and the last trial related activity.

All AEs and SA(D)E’s will be recorded in the patient’s file and in the CRF. All SADE’s will be reported as described below.

All SA(D)E’s occurring during the clinical trial must be reported by the Principal Investigator within 2 working days after becoming aware of the SA(D)E to:

- The EC
- Bimetra Clinics of the University Hospital Ghent
- The producer of the investigational medical device

For the contact details, see below.

In case the investigator decides the SAE is a USADE, Bimetra Clinics will report this to the Central EC and the CA.

In case of a life-threatening USADE the entire reporting process must be completed within 7 calendar days. In case of a non life-threatening USADE the reporting proces must completed within 15 calendar days.

The first report may be made by telephone, e-mail or facsimile (FAX).

Contact details of Bimetra Clinics:

e-mail: Bimetra.Clinics@uzgent.be

tel.: 09/332 05 00

fax: 09/332 05 20

Contact details of the National Coordinating Investigator:

Prof. dr. H. Verstraelen

e-mail: Hans.Verstraelen@ugent.be

tel.: 09/332 22 23

fax: 09/332 38 31

Contact details of the producer of the medicinal (investigational) product::

e-mail: Jody.Voorspoels@sepspharma.com

tel.: 09/261 69 01

fax: 09/261 69 20

The investigator must provide the minimal information: i.e. trial number, subject's initials and date of birth, period of intake, nature of the adverse event and investigator's attribution.

Reporting by telephone must always be confirmed by a written, more detailed report. Pregnancies occurring during clinical trials are considered immediately reportable events. They must be reported as soon as possible. The outcome of the pregnancy must also be reported.

**If the subjects are not under 24-hour supervision of the investigator or his/her staff (out-patients, volunteers), they (or their designee, if appropriate) must be provided with a "trial card" indicating the name of the investigational product, the trial number, the investigator's name and a 24-hour emergency contact number.**


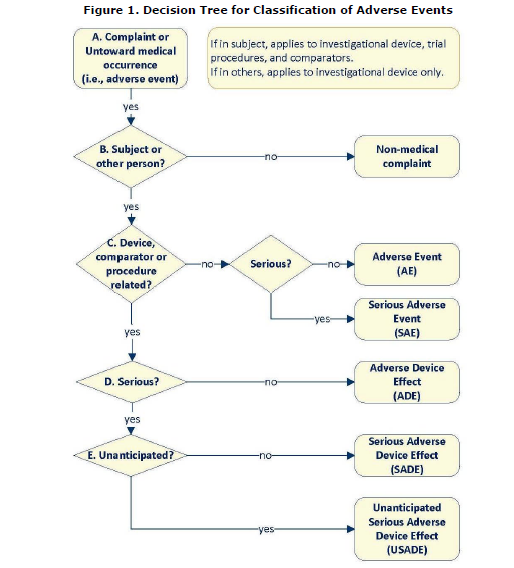


# Study analysis

## Sample size calculation

De grootte van de steekproef werd bepaald in overeenkomst met de algemeen geldende richtlijnen voor fase I studies.

## Analysis of the samples

Er is geen staalafname tijdens de studie.

## Statistical analysis

De studie is louter beschrijvend.

# Quality control and quality assurance

Quality control van de data in het CRF zal gebeuren door een persoon die niet betrokken was bij het invullen van de CRF’s; dit gebeurt door de source data met CRF te vergelijken.

# Indemnity insurance

No fault polis Universiteit Gent.

# Publication policy

De gegevens bekomen uit de studie kunnen gepubliceerd worden, zonder vermelding van personenidentificatiegegevens van de proefpersonen in een internationaal wetenschappelijk tijdschrift.

# Part II : General part of the protocol

# Independent Ethics Committee (IEC) / Institutional Review Board (IRB)

This trial can only be undertaken after full approval of the protocol and addenda has been obtained from the IEC/IRB. This document must be dated and clearly identify the protocol, amendments (if any), the informed consent form and any applicable recruiting materials and subject compensation programs approved.

During the trial, the following documents will be sent to the IEC/IRB for their review:

- reports of adverse events as described in section 10: “Adverse Event reporting”
- all protocol amendments and revised informed consent form (if any).

Amendments should not be implemented without prior review and documented approval / favorable opinion form the IEC/IRB except when necessary to eliminate an immediate hazard to trial subjects or when the change involves only logistical or administrative aspects of the trial.

Reports on, and reviews of the trial and its progress will be submitted to the IEC/IRB by the investigator at intervals stipulated in their guidelines.

At the end of the trial, the investigator will notify the IEC/IRB about the trial completion.

# ICH/GCP guidelines

This trial will be conducted in accordance with the protocol, current ICH-GCP guidelines and applicable law(s).

Good Clinical Practice (GCP) is an international ethical and scientific quality standard for designing, conducting, recording and reporting trials that involve the participation of human subjects. Compliance with this standard provides public assurance that the rights, safety and well-being of trial subjects are protected, consistent with the principles that have their origin in the Declaration of Helsinki, and that the clinical trial data are credible.

# Subject information and informed consent

Prior to entry in the trial, the investigator must explain to potential subjects or their legal representatives the trial and the implication of participation. Subjects will be informed that their participation is voluntary and that they may withdraw consent to participate at any time. Participating subjects will be told that their records may be accessed by competent authorities and by authorized persons without violating the confidentiality of the subject, to the extent permitted by the applicable law(s) and/or regulations. By signing the Informed Consent Form (ICF), the subjects or legally acceptable representatives are authorizing such access.

After this explanation and before entry to the trial, written, dated and signed informed consent should be obtained from the subject or legally acceptable representative. The ICF should be provided in a language sufficiently understood by the subject. Subjects must be given the opportunity to ask questions.

The subject or legally acceptable representative will be given sufficient time to read the ICF and to ask additional questions. After this explanation and before entry to the trial, consent should be appropriately recorded by means of either the subject's or his/her legal representative's dated signature or the signature of an independent witness who certifies the subject's consent in writing. After having obtained the consent, a copy of the ICF must be given to the subject.

In case the subject or legally acceptable representative is unable to read, an impartial witness must attest the informed consent.

Subjects who are unable to comprehend the information provided or pediatric subjects can only be enrolled after consent of a legally acceptable representative.

# Case Report Forms

The source documents are to be completed at the time of the subject’s visit. The CRFs are to be completed within reasonable time after the subject’s visit.

The investigator must verify that all data entries in the CRFs are accurate and correct. If certain information is Not Done, Not Available or Not Applicable, the investigator must enter "N.D." or "N.AV." or "N.AP", respectively in the appropriate space.

# Direct access to source data / documents

The investigator will permit trial-related monitoring, audits, IRB/IEC review, and regulatory inspection(s), providing direct access to source data/documents.

# Data handling and record keeping

The investigator and sponsor specific essential documents will be retained for at least 20 years. At that moment, it will be judged whether it is necessary to retain them for a longer period, according to applicable regulatory or other requirement(s).

# Signature page

*Investigator:*

Name: ________________________________________________

Title: ________________________________________________

Signature: ________________________________________________

Date: ________________________________________________

*Investigator:*

Name: ________________________________________________

Title: ________________________________________________

Signature: ________________________________________________

Date: ________________________________________________
