# Supplementary material for: Rationale and Safety Assessment of a Novel Intravaginal Drug-Delivery System with Sustained DL-Lactic Acid Release, Intended for Long-Term Protection of the Vaginal Microbiome
Source: PLoS One. 2016 Apr 19;11(4):e0153441. doi: 10.1371/journal.pone.0153441 (PMC4836750; doi:10.1371/journal.pone.0153441)
Supplement: S3 File — (DOC) [file pone.0153441.s003.doc]

Prof.dr. D. Matthys

Voorzitter Ethisch Comité

Kliniekgebouw 4, 1^e^ verdieping

De Pintelaan 185

9000 GENT

Gent, 19 juni 2014

Betreft: **Protocol No**.: LACRING01 – Protocol Clarification Letter 1.0

Studie naar de veiligheid van een lactaat vrijstellende vaginale ring in de profylaxe van bacteriële vaginose

EC projectnummer: 2013/088

Geachte Voorzitter,

Hieronder vindt u een aantal verduidelijkingen met betrekking tot de studie naar de veiligheid van een lactaat vrijstellende vaginale ring in de profylaxe van bacteriële vaginose. Eerst wordt de oorspronkelijke tekst weergegeven, daarnaast worden de veranderingen aangeduid in rood.

| Verwijzing protocol / ICF | Oorspronkelijk | Veranderd |
| --- | --- | --- |
| Protocol - p4/12 | Inclusion criteria  - verkeren in algemene goed gezondheid  - aanwezigheid van een normale (Lactobacillus gedomineerde) vaginale microbiota  - orale contraceptie gebruiken of bereid zijn deze op te starten in het kader van de studie | Inclusion criteria  - verkeren in algemene goede mentale en fysieke gezondheid  - aanwezigheid van een normale (Lactobacillus gedomineerde) vaginale microbiota  - orale contraceptie gebruiken of bereid zijn deze op te starten in het kader van de studie + bereid zijn om orale contraceptie door te nemen tijdens de studie zodat er geen bloeding kan worden verwacht op één van de studiedagen (screening + D1-D8)  - vrouwen tussen 18-45 jaar (uitersten inclusief) |
| Protocol - p4/12 | Exclusion criteria  - gekende ziekte  - zwangerschap  - reeds gebruik van vaginale medicatie of device  - gebruik van antibiotica in de week voorafgaand en tijdens de studie | Exclusion criteria  - gekende ziekte: hiermee worden belangrijke systeemziekten (bv. Ziekte van Crohn, reumatische aandoeningen, diabetes mellitus, …) bedoeld. Mineure aandoeningen waarvan niet verwacht wordt dat ze een invloed hebben op de studieresultaten (hooikoorts, …) vormen geen reden tot exclusie.  - zwangerschap  - vrouwen die borstvoeding geven  - Post-menopauzale vrouwen  - gebruik van vaginale medicatie of device vanaf 1 week vóór visite 2 en tijdens de studie  - gebruik van antibiotica vanaf 1 week vóór visite 2 en tijdens de studie |
| Protocol – p3/12 | Study design  Fase I studie waarbij bij 6 vrijwilligsters met een normale vaginale microbiota een onder GMP condities vervaardigde lactaat (7.5%) vrijstellende vaginale ring (zoals omschreven onder European Patent referentie EP 111 74 103.9) in de vagina wordt ingebracht en gedurende 7 dagen ter plaatse blijft. | Study design  Fase I studie waarbij bij 6 vrijwilligsters met een normale vaginale microbiota een onder GMP condities vervaardigde lactaat (7.5%) vrijstellende vaginale ring (zoals omschreven onder European Patent referentie EP 111 74 103.9) in de vagina wordt ingebracht en gedurende 7 dagen ter plaatse blijft. De studie verloopt in 2 fases. In een eerste fase zal bij 2 personen de vaginale ring worden ingebracht. Een tweede fase, met 4 proefpersonen, mag slechts worden uitgevoerd na goedkeuring van het Ethisch comité en FAGG gebaseerd op een rapport met eventuele veiligheidsrisico’s na het doorlopen van de eerste fase van de studie. |
| Protocol – p5/12 | Restrictions and prohibitions for the subjects  Proefpersonen mogen geen seksueel contact hebben vanaf 2 dagen voor de studie en tijdens de studie. Er mogen evenmin vaginale producten gebruikt worden of vaginale spoelingen worden toegepast. De proefpersonen wordt gevraagd orale contraceptie op te starten, zo dit nog niet reeds het geval is. | Restrictions and prohibitions for the subjects  Proefpersonen mogen geen seksueel contact hebben vanaf 2 dagen vóór visite 2 van de studie en tijdens de studie. Er mogen evenmin vaginale producten gebruikt worden of vaginale spoelingen worden toegepast. De proefpersonen wordt gevraagd orale contraceptie op te starten, zo dit nog niet reeds het geval is. |
| ICF – p3/8 | Er wordt gevraagd om u gedurende 1 week te onthouden van seksuele betrekkingen. | Er wordt gevraagd om zich te onthouden van seksuele betrekkingen vanaf 2 dagen (48u) vóór visite 2 tot en met de laatste visite (D8). |
| ICF – p3/8 | Op dag 1 van het onderzoek wordt dan de vaginale ring op een maandag om 9h00 ’s ochtends ingebracht | Op dag 1 van het onderzoek wordt dan de vaginale ring op een maandag vanaf 9h00 ’s ochtends ingebracht |
| ICF – p3/8 | Vervolgens kan u naar huis en wordt op dinsdag om 9h00 opnieuw een colposcopie en pH meting met pH strip verricht. | Vervolgens kan u naar huis en wordt op dinsdag vanaf 9h00 opnieuw een colposcopie en pH meting met pH strip verricht. |
| Protocol – p5/12 | De studie zal ongeveer 3 maanden duren. | De studie zal ongeveer 3 maanden duren. De tijd tussen visite 1 en 2 mag maximaal 4 weken bedragen. |
| ICF – p3/8 | Vervolgens wordt u gevraagd om gedurende één week eventuele ongewenste effecten die u zou gewaar worden in een dagboekje te noteren. | Vervolgens wordt u gevraagd om gedurende één week eventuele ongewenste effecten die u zou gewaar worden te melden aan het onderzoeksteam. |
| Protocol – p3/12 | Composition and dosing  Melkzuur (racemisch): 7.5%  Melkzuur (racemisch): 0.15 gram | Composition and dosing  L - Melkzuur: 7.5%  L - Melkzuur: 0.15 gram |

De veranderingen aan het protocol die hierboven worden beschreven, worden beschouwd als niet-substantiële wijzigingen.

Met vriendelijke groeten,

Prof. Dr. H. Verstraelen
