# Supplementary material for: Rationale and Safety Assessment of a Novel Intravaginal Drug-Delivery System with Sustained DL-Lactic Acid Release, Intended for Long-Term Protection of the Vaginal Microbiome
Source: PLoS One. 2016 Apr 19;11(4):e0153441. doi: 10.1371/journal.pone.0153441 (PMC4836750; doi:10.1371/journal.pone.0153441)
Supplement: S4 File — (DOCX) [file pone.0153441.s004.docx]

Informatiebrief voor de deelnemers aan experimenten

U wordt uitgenodigd om deel te nemen aan een onderzoek in opdracht het UZGent. Dit toestemmingsformulier bevat informatie om u te helpen beslissen of u wilt deelnemen. Neem alle tijd om dit formulier aandachtig te lezen en stel eventueel vragen aan de studiearts of het studiepersoneel.

# Titel van de studie:

Studie naar de veiligheid van een melkzuur vrijstellende vaginale ring in de profylaxe van bacteriële vaginose.

# Doel van de studie:

De vagina van de geslachtsrijpe vrouw wordt normaal behuisd door een microbiële flora bestaande uit melkzuurbacteriën, meerbepaald laktobacillen.

Soms kan een verstoring optreden van de vaginale huishouding, verdwijnen de laktobacillen grotendeels en gaan andere bacteriën massaal de vagina overgroeien, een infectie gekend als bacteriële vaginose. Dit kan gepaard gaan met een verhoogde vloeibare afscheiding en onwelriekende geur.

Deze infectie kan behandeld worden met antibiotica, maar dreigt na behandeling vaak terug te keren.

Daarom wordt naar alternatieve behandelingsstrategieën gezocht. Een veelbelovende benadering is evenwel het vaginaal inbrengen van melkzuur of lactaat, een zwak zuur dat normaal aanwezig is in de vagina.

In de jaren ’80 werden reeds verscheidene klinische studies verricht met een lactaat gel. De lactaatgels hebben evenwel verscheidene nadelen: ze vereisen frequente toediening, ze geven aanleiding tot toegenomen vaginale afscheiding en tenslotte zijn lactaatgels bij de gebruikte concentraties mogelijk irriterend voor de vagina.

Daarom ontwikkelden wij een vaginale ring die slechts om de week moet worden ingebracht en waaruit het melkzuur of lactaat traag wordt vrijgesteld in de vagina. We vermoeden daarbij dat deze ring de gezonde (laktobacillen)flora van de vagina op lange termijn beschermt en vaginale infecties zoals bacteriële vaginose zou kunnen voorkomen.

De ring werd echter nog niet bij de mens uitgetest. In een eerste fase willen we daarom bij 6 gezonde vrijwilligsters uitsluiten dat de lactaatring enig irriterend effect zou hebben op de vaginale wand.

# Wat wordt verwacht van de deelnemer?

Er kunnen redenen zijn waarom u niet mag deelnemen aan deze studie. Mogelijke redenen zijn :

- U bent niet Nederlandstalig;
- U bent een vrouw jonger dan 18 jaar;
- U bent niet in goede fysieke en mentale gezondheid;
- U bent zwanger;
- U bent in de menopauze.

Indien één van deze punten op u van toepassing is, gelieve dit dan aan de onderzoeker of één van zijn medewerkers te melden. Die zal deze en eventueel alle andere redenen waarom u niet mag deelnemen aan de onderzoeksstudie, met u bespreken. Indien u vragen heeft over het voorgaande, aarzel dan niet om ze aan de onderzoeker of één van zijn medewerkers te stellen.

# Deelname:

De deelname aan deze studie vindt plaats op vrijwillige basis.

Als u deelneemt, wordt u gevraagd het toestemmingsformulier te tekenen.

# Procedures

Indien u de mondelinge en schriftelijke informatie over de studie goed begrepen hebt en wenst deel te nemen, geeft u uw mondelinge en schriftelijke toestemming.

U ondergaat vooreerst een algemeen gynaecologisch onderzoek met zwangerschapstest en afname van een vaginaal staaltje ter uitsluiting van vaginale infecties. Indien u nog geen pil neemt, wordt u een pil voorgeschreven en gevraagd op te starten.

Op dag 1 van het onderzoek wordt dan de vaginale ring op een maandag vanaf 9h00 ’s ochtends ingebracht. U krijgt dan ook een speculumonderzoek met colposcopie om te kijken naar de vaginale wanden 1 uur, 2 uren, 4 uren, 8 uren en 24 uren na plaatsen van de ring. Verder zal er een vaginale pH meting met pH-strip om de 30 minuten gedurende de eerste 4 uren na het plaatsen van de ring en om het uur in daaropvolgende 4 uren

Vervolgens kan u naar huis en wordt op dinsdag vanaf 9h00 opnieuw een colposcopie en pH meting met pH strip verricht. Vervolgens wordt u gevraagd om gedurende één week eventuele ongewenste effecten die u zou gewaar worden te melden aan het onderzoeksteam.

Op dag 8 komt u terug naar het ziekenhuis, wordt de ring verwijderd en wordt een laatste maal een speculumonderzoek met colposcopie verricht.

Er wordt gevraagd om zich te onthouden van seksuele betrekkingen vanaf 2 dagen (48u) vóór visite 2 tot en met de laatste visite (D8).

# Risico’s en voordelen:

## risico’s

- Er zijn wellicht geen risico’s verbonden aan het onderzoek. Mogelijks veroorzaakt de ring lokaal irritatie waarna de ring onmiddellijk zal verwijderd worden.

## voordelen

Er is geen onmiddellijk voordeel voor u. De informatie die deze studie oplevert, zal evenwel andere mensen in de toekomst kunnen helpen.

U hebt het recht op elk ogenblik vragen te stellen over de mogelijke en/of gekende risico’s van deze studie. Mocht u door uw deelname toch enig nadeel ondervinden, zal u een gepaste behandeling krijgen.

Deze studie werd goedgekeurd door een onafhankelijke Commissie voor Medische Ethiek verbonden aan dit ziekenhuis en wordt uitgevoerd volgens de richtlijnen voor de goede klinische praktijk (ICH/GCP) en de verklaring van Helsinki opgesteld ter bescherming van mensen deelnemend aan klinische studies. In geen geval dient u de goedkeuring door de Commissie voor Medische Ethiek te beschouwen als een aanzet tot deelname aan deze studie.

# Kosten:

Uw deelname aan deze studie brengt geen extra kosten mee voor U, de controleconsultatie is dan uiteraard ook gratis.

# Vergoeding:

De totale vergoeding zal 250 euro bedragen, te betalen via overschrijving.

# Vertrouwelijkheid:

In overeenstemming met de Belgische wet van 8 december 1992 en de Belgische wet van 22 augustus 2002, zal u persoonlijke levenssfeer worden gerespecteerd en zal u toegang krijgen tot de verzamelde gegevens. Elk onjuist gegeven kan op uw verzoek verbeterd worden.

Vertegenwoordigers van de opdrachtgever, auditoren, het Ethisch Comité en de bevoegde overheden hebben rechtstreeks toegang tot Uw medische dossiers om de procedures van de studie en/of de gegevens te controleren, zonder de vertrouwelijkheid te schenden. Dit kan enkel binnen de grenzen die door de betreffende wetten zijn toegestaan. Door het toestemmingsformulier, na voorafgaande uitleg, te ondertekenen stemt U in met deze toegang.

Verslagen waarin U wordt geïdentificeerd, zullen niet openlijk beschikbaar zijn. Als de resultaten van de studie worden gepubliceerd, zal uw identiteit vertrouwelijke informatie blijven. Als er naar U wordt verwezen, zal dit alleen gebeuren aan de hand van codenummers.

# Letsels ten gevolge van deelname aan de studie:

De onderzoeker voorziet in een vergoeding en/of medische behandeling in het geval van schade en/of letsel tengevolge van deelname aan de studie. Voor dit doeleinde is een verzekering afgesloten met foutloze aansprakelijkheid conform de wet inzake experimenten op de menselijke persoon van 7 mei 2004. Op dat ogenblik kunnen uw gegevens doorgegeven worden aan de verzekeraar.

# Contactpersoon:

Als er letsel optreedt tengevolge van de studie, of als U aanvullende informatie wenst over de studie of over uw rechten en plichten, kunt U in de loop van de studie op elk ogenblik contact opnemen met:

Prof. dr. H. Verstraelen 09/332 37 96

Gynaecoloog van wacht 09/332 28 85

**Toestemmingsformulier**

Ik, _________________________________________ heb het document “Informatiebrief voor de deelnemers aan experimenten” met als voettekst “Informed Consent ‘LACTATE-01 studie (fase I), versie 002, 23 juni 2014” pagina 1 tot en met 5 gelezen en er een kopij van gekregen. Ik stem in met de inhoud van het document en stem ook in deel te nemen aan de studie.

Ik heb een kopij gekregen van dit ondertekende en gedateerde formulier voor “Toestemmingsformulier”. Ik heb uitleg gekregen over de aard, het doel, de duur, en de te voorziene effecten van de studie en over wat men van mij verwacht. Ik heb uitleg gekregen over de mogelijke risico’s en voordelen van de studie. Men heeft me de gelegenheid en voldoende tijd gegeven om vragen te stellen over de studie, en ik heb op al mijn vragen een bevredigend antwoord gekregen, ook op medische vragen.

Ik stem ermee in om volledig samen te werken met de toeziende arts. Ik zal hem/haar op de hoogte brengen als ik onverwachte of ongebruikelijke symptomen ervaar. Ik bevestig dat ik de toeziende arts zal inlichten over eventuele geneesmiddelen, van welke aard ook, die ik in de maand voorafgaand aan de studie heb gebruikt, momenteel gebruik of van plan ben te gebruiken, ongeacht of ze al dan niet werden voorgeschreven.

Men heeft mij ingelicht over het bestaan van een verzekeringspolis in geval er letsel zou ontstaan dat aan de studieprocedures is toe te schrijven.

Ik ben me ervan bewust dat deze studie werd goedgekeurd door een onafhankelijke Commissie voor Medische Ethiek verbonden aan het UZ Gent en dat deze studie

zal uitgevoerd worden volgens de richtlijnen voor de goede klinische praktijk (ICH/GCP) en de verklaring van Helsinki, opgesteld ter bescherming van mensen deelnemend aan experimenten.

Ik mag me op elk ogenblik uit de studie terugtrekken zonder een reden voor deze beslissing op te geven en zonder dat dit op enigerlei wijze een invloed zal hebben op mijn verdere relatie met de arts.

Men heeft mij ingelicht dat zowel persoonlijke gegevens als gegevens aangaande mijn gezondheid worden verwerkt en bewaard gedurende minstens 20 jaar. Ik stem hiermee in en ben op de hoogte dat ik recht heb op toegang en verbetering van deze gegevens. Aangezien deze gegevens verwerkt worden in het kader van medisch-wetenschappelijke doeleinden, begrijp ik dat de toegang tot mijn gegevens kan uitgesteld worden tot na beëindiging van het onderzoek. Indien ik toegang wil tot mijn gegevens, zal ik mij richten tot de toeziende arts, die verantwoordelijk is voor de verwerking.

Ik begrijp dat auditors, vertegenwoordigers van de opdrachtgever, het Ethisch Comité of bevoegde overheden, mijn gegevens mogelijk willen inspecteren om de verzamelde informatie te controleren. Door dit document te ondertekenen, geef ik toestemming voor deze controle. Bovendien ben ik op de hoogte dat bepaalde gegevens doorgegeven worden aan de opdrachtgever. Ik geef hiervoor mijn toestemming, zelfs indien dit betekent dat mijn gegevens doorgegeven worden aan een land buiten de Europese Unie. Mijn gegevens zullen wel altijd gecodeerd doorgegeven worden, waarbij mijn naam en adres geheim blijven.

Ik ben bereid op vrijwillige basis deel te nemen aan deze studie.

Naam van de vrijwilliger: _________________________________________

Datum: _________________________________________

Handtekening:

Ik bevestig dat ik de aard, het doel, en de te voorziene effecten van de studie heb uitgelegd aan de bovenvermelde vrijwilliger.

De vrijwilliger stemde toe om deel te nemen door zijn/haar persoonlijk gedateerde handtekening te plaatsen.

Naam van de persoon

die voorafgaande uitleg

heeft gegeven: _________________________________________

Datum + tijdstip _________________________________________

Handtekening:

Naam van de arts: _________________________________________

Datum + tijdstip: _________________________________________

Handtekening:
