# Supplementary material for: Rationale and Safety Assessment of a Novel Intravaginal Drug-Delivery System with Sustained DL-Lactic Acid Release, Intended for Long-Term Protection of the Vaginal Microbiome
Source: PLoS One. 2016 Apr 19;11(4):e0153441. doi: 10.1371/journal.pone.0153441 (PMC4836750; doi:10.1371/journal.pone.0153441)
Supplement: S5 File — (PDF) [file pone.0153441.s005.pdf]

STUDY NAME: DRUG13-LAC **DRUG RESEARCH UNIT GHENT** update: 13JAN15

Ring voor vaginale toediening van lactaat

	Plaatsing - verwijdering vaginale ring
Deel 1 (AN001-002)	08sep14 - 15sep14
Deel 2 Cohort 1 (AN003-004)	01dec14 - 08dec14
Deel 2 Cohort 2 (AN005-006)	10dec14 - 17dec14

CLINICAL ADVERSE EVENTS FORM

Allocation number	Adverse Event	Start Date	Start Time	Stop Date	Stop Time	Intensity	Frequency	*Relationship to study medication	Serious (no / yes)	Duration	**Outcome	***Action taken with study medication	Comments
001	runny nose	11-Sep-14	7:00	12-Sep-14	18:00	mild	intermittent	1	no		1	1	
	break through bleeding	15-Sep-14	7:00	22-Sep-14	7:00	mild	continuous	1	no		1	1	
002	post procedural hemorrhage	8-Sep-14	9:45	8-Sep-14	17:00	mild	intermittent	1	no	7 Hours & 15 Minutes	1	1	
	pressure sensation of lower abdomen	8-Sep-14	19:00	9-Sep-14	17:00	mild	intermittent	1	no	22 Hours	1	1	
	pain in jaw	13-Sep-14	20:00	ongoing	NAP	mild	continuous	1	no		4	1	
003	none												
004	none												
005	vulval irritation	10-Dec-14	11:00	10-Dec-14	13:30	mild	continous	1	no	2 Hours & 30 Minutes	1	1	procedure related
	vulval irritation	10-Dec-14	13:31	10-Dec-14	14:30	mild	intermittent	1	no	59 Minutes	1	1	procedure related
006	vaginal foreign body feeling	10-Dec-14	21:30	11-Dec-14	7:15	mild	continous	2	no	9 Hours & 45 Minutes	1	1	
	sore throat	13-Dec-14	14:00	16-Dec-14	9:30	mild	continous	1	no		1	1	
	bronchitis	16-Dec-14	9:31	11-Jan-15	8:30	moderate	continous	1	no		1	1	

*Relation to study medication	
1	definitely not related
2	probably not related
3	possibly related
4	probably related
5	definitely related

**Outcome	
1	Recovered/Resolved
2	Recovering/Resolving
3	Recovered/Resolved with Sequelae
4	Not Recovered/Not resolved
5	Fatal
6	Unknown

***Action taken with study medication	
1	None
2	Reduced
3	Interrupted
4	Discontinued
5	Increased
6	Not Applicable
7	Unknown

APPROVED BY AC+DB

1[illegible] JAN. 2015

Prof. dr. H. Verstraelen
1-46454-16-001
Vrouwenkliniek
UZ - GENT

04FEB2015
